# Prevalence of COVID-19 Pandemic-Related Distress and Suicidal Ideation in Low- and Lower Middle-Income Countries: A Systematic Review and Meta-Analysis

**DOI:** 10.1192/bjo.2024.124

**Published:** 2024-08-01

**Authors:** Usman Arshad, Mueen Abid, Imran Chaudhry, Nasim Chaudhry, Nusrat Husain

**Affiliations:** 1 Pakistan Institute of Living and Learning, Karachi, Pakistan; 2 University of Manchester, Manchester, United Kingdom; 3 Mersey Care NHS Foundation Trust, Prescot, United Kingdom

## Abstract

**Aims:**

The COVID-19 pandemic has significantly impacted healthcare systems, economies, and global health, raising concerns about its potential effects on mental health. A recent systematic review found a 40% prevalence of poor sleep quality, with 34%, 26%, and 27% prevalence for psychological distress, depression, and anxiety. The systematic review investigated COVID-19-related stress, suicidal ideation, and self-harm thoughts among low- and lower-middle-income countries (LLMICs).

**Methods:**

We search four electronic databases (PsycINFO, Medline, Embase, and PubMed). Quantitative studies, including both published and grey literature, from LLMICs focused on the prevalence of suicidal ideation or psychological distress during COVID-19 were included. Qualitative studies, non-English studies without full-text English translation, meta-analysis, commentary, books, and discussion articles were excluded.

**Results:**

1157 titles and abstracts were screened for inclusion and exclusion, resulting in 79 full-text articles. After full text screening, 11 articles were included. In Bangladesh, 12.8% of university students reported suicidal ideation (SI), while 19% of young adults had SI, and 18.5% reported suicidal planning. In addition to this, in Iran, 12.8% of pregnant women and in the Philippines, 24.9% of the general population reported SI. Mental health conditions like depression and anxiety, female gender, younger age groups, economic loss or financial stress, fear of COVID-19 infection, lack of social support, family problems, lower education levels, smoking, and substance use are identified as risk factors. Moreover, anosmia and dysgeusia symptoms were associated with a 30–80% increased risk of transitioning to suicidal ideation or depression in India. A study from Nepal reported a 44% increase in suicide attempts during lockdown compared with pre-pandemic periods in Nepal.

**Conclusion:**

The findings of this review suggest that the impact of the COVID-19 pandemic on mental health in LLMICs is substantial. In addition to the increased risk of SI and suicide attempts, there was a significant rise in depression and SI associated with anosmia and dysgeusia symptoms. These results underscore the urgent need for increased psychosocial support in LLMICs to address the growing mental health burden caused by the pandemic. Moreover, understanding the long-term effects of the pandemic is crucial for developing effective interventions and support systems. Further research is needed to examine the lasting impact of the pandemic on mental well-being and identify future strategies.

